# The sitting vs standing spine^☆^

**DOI:** 10.1016/j.xnsj.2022.100108

**Published:** 2022-03-02

**Authors:** Christos Tsagkaris, Jonas Widmer, Florian Wanivenhaus, Andrea Redaelli, Claudio Lamartina, Mazda Farshad

**Affiliations:** a Department of Orthopedics, Balgrist University Hospital, Zurich, Switzerland; b Spine Biomechanics, Department of Orthopaedics, Balgrist University Hospital, Zurich, Switzerland; c GSpine4 - I.R.C.C.S. Istituto Ortopedico Galeazzi, Milan, Italy

**Keywords:** Sitting radiographs, Seated imaging, Spine, spinal fusion, Spine surgery, EOS imaging, sedentary lifestyle

## Abstract

**Background:**

Planning of surgical procedures for spinal fusion is performed on standing radiographs, neglecting the fact that patients are mostly in the sitting position during daily life. The awareness about the differences in the standing and sitting configuration of the spine has increased during the last years. The purpose was to provide an overview of studies related to seated imaging for spinal fusion surgery, identify knowledge gaps and evaluate future research questions.

**Methods:**

A literature search according to the Preferred Reporting Items for Systematic Reviews and Meta-Analysis (PRISMA) extension for Scoping Reviews (PRISMASc) was performed to identify reports related to seated imaging for spinal deformity surgery. A summary of the finding is presented for healthy individuals as well as patients with a spinal disorder and/or surgery.

**Results:**

The systematic search identified 30 original studies reporting on 1) the pre- and postoperative use of seated imaging of the spine (n=12), 2) seated imaging of the spine for non – surgical evaluation (n=7) and 3) seated imaging of the spine among healthy individuals (12). The summarized evidence illuminates that sitting leads to a straightening of the spine decreasing thoracic kyphosis (TK), lumbar lordosis (LL), the sacral slope (SS). Further, the postural change between standing and sitting is more significant on the lower segments of the spine. Also, the adjacent segment compensates the needed postural change of the lumbar spine while sitting with hyperkyphosis.

**Conclusions:**

The spine has a different configuration in standing and sitting. This systematic review summarizes the current knowledge about such differences and reveals that there is minimal evidence about their consideration for surgical planning of spinal fusion surgery. Further, it identifies gaps in knowledge and areas of further research.

## Introduction

Spinal fusion surgery has increased in frequency over the last two decades. Complications rates remains high affecting between 29 and 62% of individuals undergoing this type of surgery [1], [2], [3], [4]. The same upward trend applies to the relevant healthcare expenses and disability [5].

Thorough preoperative planning has a major potential to decrease the likelihood of complications and tailor the treatment to the condition and the needs of the patient. A wealth of techniques has been employed to optimize and personalize preoperative planning. These include sagittal alignment parameters' mapping (sagittal vertical axis – SVA, pelvic tilt – PT, pelvic incidence and lumbar lordosis mismatch - PI-LL mismatch, T1 pelvic angle - TPA) assistive planning software, 3D spinal anatomy reconstruction of biplanar radiographic images and 3D printing of patient – specific instrumentation [6], [7], [8]. To date, preoperative planning has been greatly based on standing radiographies – with supine computed tomography (CT) and magnetic resonance imaging (MRI) as an adjunct.

This stated, it appears that conventional imaging and subsequent planning have ignored the fact that people spend a significant part of the daytime sitting. Sedentary behavior is a complex phenomenon involving physiological and kinematic adaptation of the body and oftentimes associated with musculoskeletal, cardiovascular and metabolic implications. Numerous sitting patterns have been documented across different population groups and cultures [9], [10], [11]. According to a recent study, nearly 20% of the population of Europe spends more than 7.5 hours per day on a chair [12]. People in North Europe sit more, with an average of 6,5 hours daily, while people in southern and eastern Europe sit for at least 3 hours daily. A number of factors including urbanization and the shift of the labor markets towards white collar professions have promoted sedentary lifestyle among almost all age groups [13,14]. Particularly since the beginning of the COVID-19 pandemic in early 2020, lockdowns and home – office mandates have significantly increased sitting time and its musculoskeletal implications in all population groups including children and young adults [15]. Therefore, sitting radiographs and adjunct imaging acquired at a seated position should potentially be integrated in spinal fusion preoperative planning. To achieve so, a robust body of evidence investigating the benefits and challenges of sitting radiographies in spinal fusion needs to be created. The first step in this regard is to map the available evidence and identify knowledge gaps.

**Aim** To provide a systematic overview of the available evidence on differences on the spinal configuration in sitting and standing, identify research gaps and discuss their implications in future research and clinical practice.

## Methods

To identify relevant peer reviewed publications and grey literature the authors searched PubMed-Medline, Web of Science, Cochrane Library‒Cochrane Central Register of Controlled Trials (CENTRAL) and Clinicaltrials.gov until September 10, 2021. The reference lists of the selected sources were also hand – searched to identify potentially relevant resources. The authors used the search terms: “sitting radiographs”, “seated imaging”, “spine surgery”, “spine fusion [MeSH]” in combination with Boolean operators (AND, OR), when appropriate. Studies were included if they fulfilled all the following eligibility criteria: (1) ongoing or published clinical studies and systematic reviews reporting the use of sitting imaging in spine deformity surgery, (2) prospective and retrospective, human and animal studies reporting on the same, and (3) cohort or cross-sectional studies. A study was excluded if it met at least one of the following criteria: (1) non-English or German publication language, (2) study types: opinion articles and perspectives. No sample size restriction was applied when screening for eligible studies. Disputes in the selection of relevant studies were discussed between the primary authors and a senior author until a consensus was reached. The literature was searched and reported according to the Preferred Reporting Items for Systematic Reviews and Meta-Analysis (PRISMA) extension for Scoping Reviews (PRISMASc). IBM SPSS Statistics 26 was used for statistical analysis of the included studies' characteristics.

## Results

**Fig. 1:**
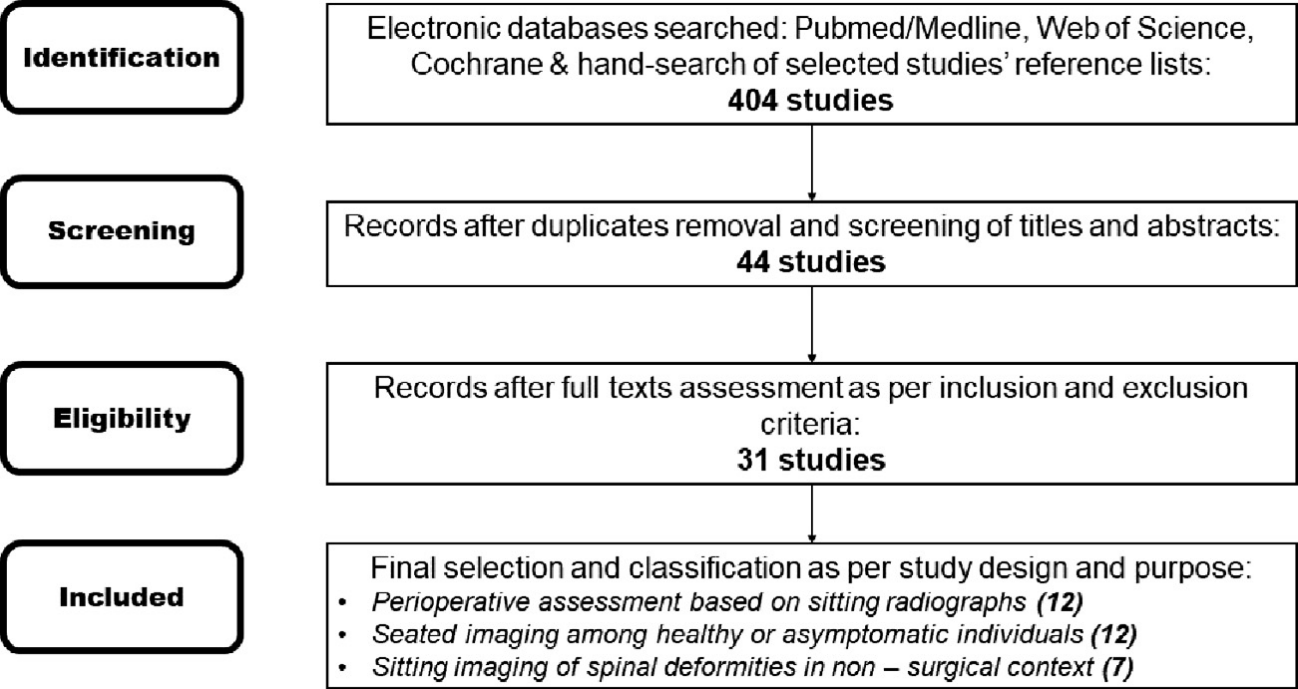
Literature search flow diagram.

The initial search including hand – searching of reference lists yielded 404 studies. After removing duplicates and screening titles and abstracts the authors evaluated the full texts of 44 studies. As per inclusion and exclusion criteria 31 studies were eligible for inclusion. A literature search flow is presented in Fig. 1.

The authors divided the studies into 3 categories based on the study design and purpose; namely based on the use of seated imaging in perioperative (preoperative and/or postoperative settings), in non – operative settings and in the assessment of healthy individuals or individuals without a history of spine condition. Most studies were observational (retrospective, cross – sectional or prospective). Approximately one third were related to the perioperative assessment of patients, approximately one third of studies assessed spinal conditions by means of sitting imaging irrespective of surgery and the remaining evaluated the radiological characteristics of the sitting spine in healthy individuals (not diagnosed with a spine condition). The majority of the studies were based on plain radiographs, while few studies used EOS radiographs (3) or positional MRI (5). The majority of studies focused on the lower segments of the spine (lumbar, sacral), with a limited number assessing the thoracic spine or global spinal alignment. Only one study focused exclusively on the cervical spine. The majority of relevant studies have been published after 2018, with only two studies being published before 2010. All the studies, apart from Moon et al. 2018 [16] evaluated subjects on a common natural sitting position comparing it with conventional standing lateral and/or sagittal radiographies. Few studies included more seated position variants in their analysis; namely erect sitting [17], floor sitting [16,18], sitting on a kneeling chair, sitting on a chair with back support, sitting on 90° angled chair, sitting on chair with anterior support, sitting on stool, sitting cross-legged [19], kneel sitting [16], anteflexed sitting [20], upright sitting [20,21], seated flexion [21,22], seated right and left axial rotation [21], reclined and forward inclined sitting [23]. An overview of the included studies' and subjects' characteristics is presented in Table 1.

**Table 1:** Overview of the included studies' and subjects' characteristics.

Study	Date	Study type	Context	Sample	Spine conditions	Spine segment	Imaging type	Seated position
Yoshida et al.	2020	Observational clinical study	Perioperative imaging	113	Adult spinal deformity	Thoracic, Lumbar, sacral	Sitting XR	Natural sitting
Hey et al.	2020	Observational clinical study	Perioperative imaging	120	Low back pain	Lumbar, sacral	Sitting XR	Natural sitting
Zhao et al.	2019	Observational clinical study	Perioperative imaging	36	Thoracolumbar kyphosis	Lumbar, sacral	EOS	Natural sitting
Janjua et al.	2018	Observational clinical study	Perioperative imaging	20	Thoracolumbar deformity	Thoracic, Lumbar, sacral	Sitting XR	Natural sitting
Zhu et al.	2018	Observational clinical study	Perioperative imaging	44	Idiopathic thoracic scoliosis	Thoracic, Lumbar, sacral	Sitting XR	Natural sitting
Chiu et al.	2018	Observational clinical study	Perioperative imaging	28	Osteoporotic thoracolumbar fractures	Lumbar, sacral	Sitting XR	Natural sitting
Hey et al.	2017	Observational clinical study	Perioperative imaging	70	Low back pain	Thoracic, Lumbar, sacral	Sitting XR	Natural sitting
Hey et al.	2017	Observational clinical study	Perioperative imaging	58	Low back pain	Lumbar, sacral	EOS	Natural sitting
Vaughn et al.	2014	Observational clinical study	Perioperative imaging	26	Idiopathic scoliosis	Thoracic, Lumbar, sacral	Sitting XR	Natural sitting
Siddiqui et al.	2005	Observational clinical study	Perioperative imaging	12	Symptomatic lumbar spinal stenosis	Lumbar, sacral	MRI	Natural sitting
Zhou et al.	Ongoing	Clinical trial	Perioperative imaging	200	Adult degenerative scoliosis	Lumbar, sacral	Sitting XR, MRI	Natural sitting
Sun et al.	2020	Observational clinical study	Postoperative imaging	63	Lumbar degeneration	Lumbar, sacral	Sitting XR	Natural sitting, Erect sitting
Nishida et al.	2020	Observational clinical study	Physiological imaging	113	Healthy	Global spinal alignment	Sitting XR	Natural sitting
Maekawa et al.	2019	Observational clinical study	Physiological imaging	253	Healthy	Lumbar, sacral	Sitting XR	Natural sitting
Berry et al.	2019	Observational clinical study	Physiological imaging	13	Healthy	Lumbar, sacral	MRI	Natural sitting, Seated right axial rotation, Seated left axial rotation
Chevilotte et al.	2018	Observational clinical study	Physiological imaging	15	Healthy	Lumbar, sacral	Sitting XR	Natural sitting, Upright sitting, Seated flexion
Suzuki et al.	2018	Observational clinical study	Physiological imaging	25	Healthy	Lumbar, sacral	Sitting XR	Natural sitting, Anteflexed sitting, Upright sitting
Moon et al.	2018	Observational clinical study	Physiological imaging	16	Healthy	Lumbar, sacral	Sitting XR	Floor sitting, Kneel sitting
Alamin et al.	2018	Observational clinical study	Physiological imaging	20	Healthy	Lumbar, sacral	Sitting XR	Natural sitting, Sitting on a kneeling chair, Sitting on a vertical angled chair, Sitting on a chair with back support, Sitting on a chair with anterior support, Sitting on stool, Cross leged sitting
Suzuki et al.	2016	Observational clinical study	Physiological imaging	73	Healthy	Lumbar, sacral	Sitting XR	Natural sitting
Cho et al.	2015	Observational clinical study	Physiological imaging	30	Healthy	Lumbar, sacral	Sitting XR	Natural sitting
Bae et al.	2012	Observational clinical study	Physiological imaging	30	Healthy	Lumbar, sacral	Sitting XR	Natural sitting, Floor sitting
Endo et al.	2012	Observational clinical study	Physiological imaging	16	Healthy	Lumbar, sacral	Sitting XR	Natural sitting
Baumgartner et al.	2012	Observational clinical study	Physiological imaging	6	Healthy	Global spinal alignment	MRI	Upright sitting, Reclined Sitting, Forward inclined sitting
Zhou et al.	2021	Observational clinical study	Non-surgical evaluation	62	Symptomatic spondylolisthesis (lumbar degeneration)	Lumbar, sacral	Sitting XR	Natural sitting
Inoue et al.	2021	Observational clinical study	Non-surgical evaluation	23	Lumbar spondylosis	Lumbar, sacral	Sitting XR	Natural sitting
Sielatycki et al.	2021	Observational clinical study	Non-surgical evaluation	70	Low back pain	Lumbar, sacral	Sitting XR	Natural sitting, Seated flexion
Kusakabe et al.	2019	Observational clinical study	Non-surgical evaluation	108	Spinal degeneration	Cervical	Sitting XR	Natural sitting
Rouissi et al.	2016	Imaging protocol	Non-surgical evaluation	36	Neuromuscular scoliosis	Lumbar, sacral	EOS	Natural sitting
Bouloussa et al.	2015	Imaging protocol	Non-surgical evaluation	41	Neuromuscular scoliosis	Lumbar, sacral	EOS	Natural sitting
Karadimas et al.	2006	Observational clinical study	Non-surgical evaluation	30	Low back pain	Lumbar, sacral	MRI	Natural sitting

### Implications in healthy adults

Studies in healthy individuals comparing a standard standing and sitting position reported that sitting affects predominantly the thoracolumbar spine, from T10-T11 to L5-S1 [23]. Seated position leads to an increase in cervical lordosis (CL) and decrease in thoracic kyphosis (TK) [24] and approximately 50% decrease in lumbar lordosis (LL) [20,[24], [25], [26], [27], [28]. The extent of the LL decrease varied significantly with age [24]. More specifically, the decrease in LL was significantly (by approximately 15%) reduced among the middle aged and elderly in comparison to young adults [25,26]. LL was positively correlated with thoracic kyphosis [24,27]. The sacral slope (SS) is also decreased by up to 50% when sitting [20,[24], [25], [26], [27], [28] and again the SS decrease among the elderly is reduced by approximately 15% in comparison to young adults [25,26]. On the contrary, the PT is increased up to 25% in seated position [20,[24], [25], [26], [27], [28]. The PI remains constant [27,28]. Sitting leads to a retroversion of the pelvis [20], but lumbopelvic mobility appears poor among the elderly [25]. The realignment of the spine in sitting position leads to greater loading towards the intervertebral discs (IVDs) [28], translocating the nucleus pulposus posteriorly [21]. Details regarding the changes in spinal alignment are presented in Table 2. Although all studies are consistent in terms of the decrease or increase of certain spinal alignment parameters, there is a numerical variability which can be associated with the mixed age groups included and the lack of a strict definition for the standard sitting position among others.

**Table 2:** Lumbar Lordosis (LL), Sacral Slope (SS) and Pelvic Tilt (PT) in standing and sitting position, all values are expressed in (°).

LL Stand.			LL Sit.	SS Stand.	SS Sit.	PT Stand.	PT Sit.	Reference
36.2±12.1			0.7±26.3 (↓)	35.8 ± 21.8	16.6± 9.38 (↓)	14.4±7.27	65.17± 8.24 (**↑**)	Nishida et al. 2020
Young adults		49.3 ± 14.2	23.3 ± 13.4 (↓)	34.6 ± 7.7	18.1 ± 10.1 (↓)	19.7 ± 16.4	32.5 ± 12.7 (**↑**)	Maekawa et al. 2019
Middle aged		40.8 ± 11.5	24.9 ± 16.2 (↓)	31.3 ± 8.5	18.8 ± 10.1 (↓)	22.2 ± 15.1	33.3 ± 14.0 (**↑**)	
Elderly		42.1 ± 14.1	27.1 ± 14.8 (↓)	31.6 ± 8.9	20.1 ± 9.6 (↓)	24.3 ± 15.8	33.2 ± 14.7 (**↑**)	
54.8 ± 9.8			15.9° ± 14.6 (↓)	37.1 ± 6.3	11.3 ± 10.8 (↓)	12.1± 6.3	37.7± 10.4 (**↑**)	Chevillote et al. 2018
31.9 ± 10.4			7.9 ± 10.8 (↓)	35.9 ± 8.7	14.9 ± 11.7 (↓)	7.7 ± 9.5	31.5 ± 8 (**↑**)	Suzuki et al. 2018
Young adults	31.3 ± 10.4		15.5 ± 10.1 (↓)	36.4 ± 7.2	19.0 ± 9.7 (↓)	10.3 ± 7.3	27.6± 10.5 (**↑**)	Suzuki et al. 2016
Elderly	26.6 ± 12.8		16.0 ± 13.9 (↓)	32.6 ± 8.6	21.2 ± 10.9 (↓)	15.0 ± 7.3	27.5 ± 10.9 (**↑**)	
33.3 ± 11.2			16.7 ± 11.2 (↓)	37.2 ± 7.1	18.5 ± 10.9 (↓)	9.9 ± 7.4	28.2± 10.8 (**↑**)	Endo et al. 2012
X			24.7 ± 8.3 (↓)	X	X	X	X	Baumgartner et al. 2012
**Mean LL decrease**			56% **↓**	**Mean SS decrease**	49% ↓	**Mean PT increase**	58% **↑**	

**Fig. 2:**
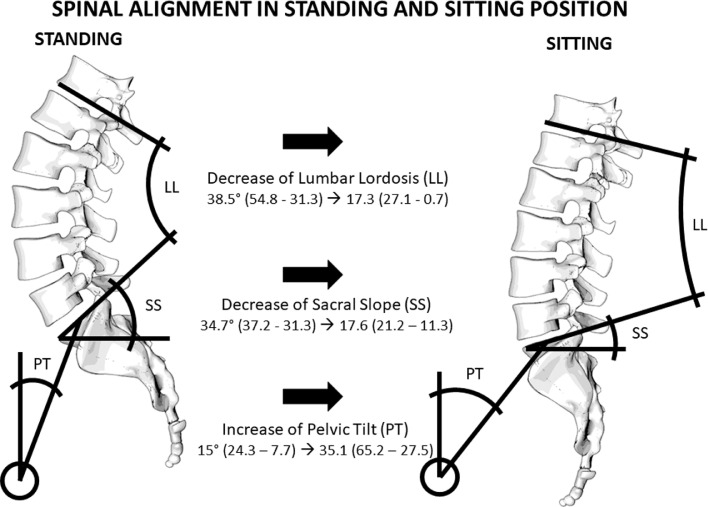
Graphical overview of spinal alignment in sitting and standing position.

Studies that compared more variations of sitting provide further insights. It seems that kneel sitting [16] and sitting on a chair with back support [29] do not differ significantly from standing in terms of spinal alignment. However, LL, SS and PT were significantly different in cross legs sitting. LL was decreased by up to 75% in comparison to standing and 40% in comparison to chair sitting, SS was decreased by up to 63% and 33%, and PT was increased by 64% and 44% respectively [16]. Floor sitting also leads to a significant decrease in LL, approximately 74% and 57% in comparison to standing and sitting accordingly. Segmental lordosis is greatly altered in the L4-S1, where it decreases by 60% in chair sitting and by approximately 70% in floor sitting [18]. Similar decrease of the L4-5 segmental angulation, ranging between 60-70%, was measured in hard-back-chair and stool sitting. Posterior disc heights were increased by approximately 10% in L1-L2. The difference in disc height in other lumbar segments did not change significantly between natural sitting, sitting on a kneeling chair, sitting on a vertical angled chair, sitting on a hard- back chair, sitting on a chair with anterior support, sitting on stool, and cross leged sitting [19]. An overview of the alterations in spinal alignment between standing and sitting is provided in Fig. 2.

### Clinical implications in patients

Studies involving patients pre- and/or postoperatively or regardless of surgical intervention provide useful information regarding spinal alignment in standing position in a number of spinal deformities and its potential implications on spine surgery.

#### Fusion surgery

A number of studies included patients who underwent (or were eligible to undergo) fusion surgery. Hey and colleagues (2017) compared spinal alignment between standing and sitting in patients with low back pain of various etiologies and noticed forward SVA displacement, superior movement of the apex vertebra towards the lumbar curve and inferior towards the thoracic curve by one vertebral level. There were significant differences with a TK decrease by 30%, LL decrease by 50%, SS by 40%, PT increase by 53% and the thoracolumbar junctional angle tended to become less kyphotic and more lordotic. Although these observations are consistent with the ones on healthy individuals, a slight reduction in SS decrease (9%) and in PT increase (8%) was observed [30], implying that patients undergone lumbar fusion are more likely to have residual lordosis, particularly at the lower lumber spine, in natural sitting position [17].

**Fig. 3:**
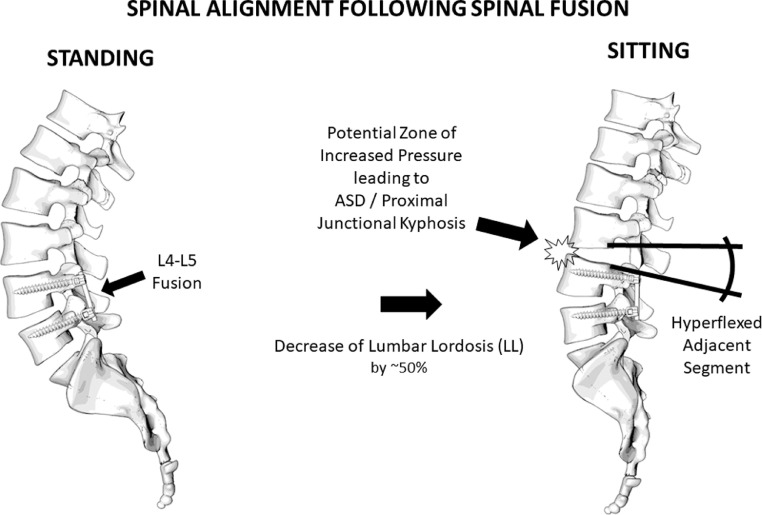
Graphical overview of spinal alignment in sitting and standing posture in patients undergone spinal fusion.

The second study of the same group provided more clarifications by investigating the spinal ROM in different postures including slump sitting. It appears that the greatest ROM of the lumbar spine is achieved in slump sitting, particularly in the L4-L5 segment, whose mobility reaches approximately 50%. In this position, L1-L4 may even become kyphotic [31]. Pre- to postoperative changes in kyphosis can be predicted by the difference between sitting to standing radiographs. To date, the most reliable predictor is a plumb line distance between the upper instrumented vertebra (UIV) and the C2 with a cutoff value of 11,5 cm [7,32]. Considering these together with the fact that extended L1-L5 fusion would decrease lumbar flexion by 47.6° and lumbar extension by up to 5.9°, it becomes evident that lower lumbar fusion can lead to a malalignment of their adjacent segments. Failure to address so with suitable spine instrumentation can precipitate adjacent segment degeneration (ASD) [31]. An overview of the impact of fusion surgery on spinal alignment is provided in Fig. 3.

#### Scoliosis

Five studies have focused on scoliosis (adult degenerative scoliosis, idiopathic thoracic scoliosis and neuromuscular scoliosis). Rouissi et al. (2017) and Bouloussa et al. (2016) described a protocol for EOS imaging in neuromuscular scoliosis; their results were oriented towards feasibility and satisfaction measures and did not have direct reference to surgery [33,34]. The studies of Vaughn, Chiu, Hey and their colleagues assessed preoperative and, in some cases, postoperative spine seated imaging. Preoperative sitting imaging in patients with idiopathic scoliosis reveals a decrease in TK, LL, SS [35], [36], [37]. The decrease in TK appears reduced in comparison to healthy or non - scoliotic individuals (approximately 10% instead of 30%). Postoperative changes should also be considered. Following posterior thoracic fusion in patients with idiopathic thoracic scoliosis a significant reduction in LL and SS decrease (9.7% and 5.7% in contrast to 42.1% and 31.1% preoperatively respectively) and a significant reduction in PT increase (39.0% in contrast to 193.6%) from standing to sitting occur [36].

The shape of the spine can affect this type of predictions according to a study of Hey and colleagues (2020) assessing the implications of a predilection towards S- or C-shaped spine to spine realignment surgery. Although, the LL decreases by an average of 75% in both types of spinal deformity when sitting, LL can be up to 20% larger in S-shaped spines in sitting position. SS differs significantly between the two types of spine alignment and while in S-shaped spines SS tends to decrease by 75% in sitting, in C-shaped spines the angle can be retroverted (from 32 degrees to - 0.9 degrees). PT increased by 68% in C-shaped spines and by 58% in S-shaped spines.

#### Osteoporotic fractures

Sitting radiographs can also be useful in vertebroplasty for osteoporotic fractures. Zhu et al. (2018) evaluated dynamic stress mobility radiographs (including sitting radiographs) in an attempt to predict the vertebral height restoration, kyphosis correction, and cement volume required in vertebroplasty for osteoporotic thoracolumbar vertebral fractures with intravertebral cleft. Calculating the supine stress versus sitting difference (SSD) enabled the researchers to predict that following vertebroplasty the kyphotic wedge endplate angle (WEPA) and the regional kyphotic angle (RKA) would decrease by 50% [38].

### Non-surgical insights

Additional insights can be traced in studies that did not report on surgical associated sitting imaging. The combination of sitting radiographs with supine sagittal MRIs is more accurate in revealing high vertebral slip percentages in comparison to standing radiographs [39]. Seated imaging is also more accurate in revealing kyphosis, particularly in the lower lumbar segments (L4-S1) [22]. Sitting does not significantly affect the cervical spine, unless there is significant vertebral imbalance leading to decreased cervical lordosis (CL) and large LL-PI mismatch that leads to increased CL [39]. Nevertheless, sitting radiographs were not as accurate as the lateral decubitus position for the assessment of spinal instability Inoue et al. (2021) [40]. Nonetheless, seated MRI was more accurate in detecting degeneration associated decrease in end – plate angles than supine or standing imaging. Seated MRI also revealed an increase in the anterior and middle disc heights by approximately 16% when sitting, regardless of the degree of lumbar degeneration [41].

## Discussion

The spine has a different configuration in standing and sitting. Sitting tends to straighten the spine decreasing TK, LL and SS up to approx. 50% and increasing PT by 50% as well. After spinal fusion, the upper adjacent segment needs to compensate for the decrease in LL and therefore it becomes more kyphotic while sitting. The hyperkyphosis of the adjacent segment might result in the formation of a zone of increased compression in the anterior spine and increased tension on the posterior spine. This phenomenon, which is known as proximal junctional kyphosis and is an ASD feature per se, sheds light to potential biomechanical ASD risk factors [32,^42^,43]. These include increased intradiscal pressure, posterior translocation of the nucleus pulposus, altered angular mobility at the proximal kyphotic level, adaptation of the paraspinal muscles and ligaments to the new loading conditions and subsequent injury of these structures. Structural damage as a result of this pathomechanical cascade can alter loading mechanics, severe the local vasculature and disc nutrition and trigger reactive inflammation [44]. Cumulatively, these can accelerate the degeneration of the involved level. The amount of this phenomenon might be dependent on the fusion angle, the quality of instrumentation and the different patterns of tissue damage caused by anterior and posterior fusion techniques (laminectomy, iatrogenic muscle damage, ligament rupture, soft tissue defect). Sitting radiographs could therefore be used to predict the postoperative reciprocal change and might influence surgical planning (alteration of fusion angle, soft landing techniques etc). Further, these findings might lead the surgeon to advise patients against floor sitting after fusion surgery, given that it leads to adjacent segments hyperflexion [18].

In patients with scoliosis, TK, LL, SS are also significantly decreased in sitting position, but the extent of decrease is reduced by approximately 10% (JJ Vaughn & RM Schadjacwend, 2014). These parameters can be decreased up to an additional 10% after surgery [36]. Their spine is less straightened in sitting position. This means that preoperative planning with sitting radiographs should be potentially different in scoliosis surgery compared to other types of spinal fusion. Further research is needed to find the relevance of these finding for management of scoliotic deformities.

The limited evidence about sitting radiographs and relevant planning in spinal surgery has multiple and interconnected causes. First and foremost, the lack of sufficient knowledge about mechanical stresses imposed to the spine during sitting and their potential impact on vertebrae, IVDs, spinal joints and ligaments hinders the development of sitting imaging techniques [19]. Further, erect radiographs have technical advantages over radiographs acquired in sitting position. In particular, they reflect the weight – bearing condition of the spinal structures, they can be obtained more easily from children, overweight and obese individuals [45]. Defining a standard sitting imaging position, training radiologists to obtain and interpret such radiographs and modifying the existing imaging facilities accordingly would be challenging, costly and time-consuming. Lastly, it is only during the last few years that sitting- and therefore sitting imaging - gained relevance because the combined effect of urbanization and marked increase in the non – manual labor force significantly increased the time that individuals spend sitting [10,46].

The exponential increase in sitting and its musculoskeletal sequelae during the COVID-19 pandemic enhances the relevance of this review further. Since early 2020, numerous studies have stressed that low back pain and sagittal imbalance has become quite more frequent in individuals of all ages and particularly students and young workers [15,[47], [48], [49], [50], [51]. Oftentimes, these individuals enter a vicious circle, where they seek relief from pain in sitting, but bad sitting postures only deteriorate their pain and the underlying spinal condition [52]. This is expected to increase the demand for spine surgery among younger or middle – aged individuals in the foreseeable future. Given the demanding lifestyle and the increased life – expectancy of this patients' group, decreasing the rate of complications and revision surgery is a dire need. Sitting imaging has a major potential to address this need, because sitting will constitute both a pathogenetic mechanism and a significant lifestyle factor in this population. Furthermore, sitting imaging research will help attract funding and investments to counter the damage that the pandemic has inflicted to elective spine surgery and related research and entrepreneurship [53], [54], [55]. Henceforth, this review also prompts the need for more preclinical and clinical research in the field.

### Limitations and future research

Even if we were not able to perform a lack of risk of bias analysis and metanalysis due to the heterogeneity of the included studies and we did not include studies published in languages different than English and German, we could identify following areas of future research.

At preclinical level, more attention should be given to the loading alterations in sitting position, the effect of sitting on muscles, tendons, proprioceptive networks and reflexes involved in spine biomechanics and the finite elements' behavior and pathogenetic adaptation – coping mechanisms. At clinical level, it is important to delve into instrumentation fatigue and screw loosening associated with sitting posture and relevant radiological markers, different instrumentation techniques and their biomechanical effect on the fused and adjacent segments in sitting vs standing, the contribution of sitting to the development of particular spinal fusion complications, the standard sitting position which emulates the most frequent sitting patterns and can be used by radiologists in the future and the sensitivity and specificity of specific seated imaging modalities (radiographs, EOS, MRI) in the form of indications for pre- and postoperative imaging in specific conditions and operations.

Subsequently, epidemiological studies should seek sitting – associated risk factors for spinal surgery failure among patients and investigate whether preoperative planning based on sitting imaging is more effective in particular population or patients' groups depending on their age, gender, weight, occupation or disease history. Research in population health and epidemiology should also provide input regarding the sitting patterns in modern societies through cross sectional and participant observation studies and educate the public on healthy sitting behaviors.

Certainly, the integration of seated imaging in clinical practice will require meta-research to generate specific guidelines and feasibility studies to assess the implementation, financial and educational aspects of the matter. Proof of concept studies are needed to develop and validate 1) a standard behavioral assessment of individual sitting patterns documented with sitting imaging in the most common sitting positions *(kathistography)* and 2) personalized preoperative planning based on the evaluation of the patient's sitting pattern *(lifestyle adjusted spine surgery).*

## Conclusion

Seated imaging has a major potential to improve planning of spinal surgery. Existing studies have shown a number of spinal alignment alterations associated with the straightening of the spine, particularly the lower segments, in sitting position. Sitting decreases TK, LL and SS by up to 50% and increases PT at the same rate in both healthy individuals and patients. Failing to take these into account when performing spinal fusion or choosing instrumentation adjusted to standing radiographs may have considerable implications.

## Funding Disclosures

No funding was obtained for this study.

## Declaration of Competing Interest

The authors declare that they have no known competing financial interests or personal relationships that could have appeared to influence the work reported in this paper

## Appendix Supplementary materials

Image, application 1

Image, application 2

Image, application 3
