# Supplementary material for: The sitting vs standing spine
Source: N Am Spine Soc J. 2022 Mar 2;9:100108. doi: 10.1016/j.xnsj.2022.100108 (PMC8924684; doi:10.1016/j.xnsj.2022.100108)
Supplement: Supplementary file 3 [file mmc3.docx]

**THE SITTING VS STANDING SPINE**

Christos Tsagkaris MD^1,2^, Jonas Widmer PhD^1,2^, Florian Wanivenhaus MD^1^, Andrea Redaelli MD^3^, Prof. Claudio Lamartina MD^3^, Prof. Mazda Farshad MD, MPH^1^

^1^ Department of Orthopedics, Balgrist University Hospital, Zurich, Switzerland

^2^ Spine Biomechanics, Department of Orthopaedics, Balgrist University Hospital, Zurich, Switzerland

^3^ GSpine4 - I.R.C.C.S. Istituto Ortopedico Galeazzi, Milan, Italy

**ORCID IDs:** Christos Tsagkaris: <https://orcid.org/0000-0002-4250-574X>; Jonas Widmer: <https://orcid.org/0000-0003-3676-008X>; Florian Wanivenhaus <https://orcid.org/0000-0002-1735-4951>; Andrea Redaelli: <https://orcid.org/0000-0003-2907-6263>; Claudio Lamartina: <https://orcid.org/0000-0002-8790-7064>; Mazda Farshad: <https://orcid.org/0000-0002-7190-1127>

**Corresponding author:**

Prof. Mazda Farshad MD, MPH

Balgrist University Hospital

Department of Orthopedics, University of Zurich

Balgrist Campus, Lengghalde 5

CH - 8008 Zurich

Tel +41 44 386 3004

Fax +41 44 386 3009

[mazda.farshad@balgrist.ch](mailto:mazda.farshad@balgrist.ch)

**Funding Disclosures:** No funding was obtained for this study

**Conflict of interest statement:** The authors declare that they have no known competing financial interests or personal relationships that could have appeared to influence the work reported in this paper

**Summary Sentence:** Planning of surgical procedures for spinal fusion is performed on standing radiographs, neglecting the fact that patients are mostly in the sitting position during daily life. Increasing awareness regarding the different configuration of the spine in standing and sitting position, can help improve surgical planning and outcomes
